# N-of-1 tissue activation modeling reveals regional subthalamic stimulation predicting verbal fluency decline in Parkinson disease

**DOI:** 10.1007/s00702-026-03150-y

**Published:** 2026-04-06

**Authors:** Mary J. Robinson, Kelvin L. Chou, Taylor R. Schmitt, Parag G. Patil, Karlo A. Malaga

**Affiliations:** 1 Department of Biomedical Engineering, Bucknell University, Lewisburg, PA USA; 2 Department of Neurology, University of Michigan, Ann Arbor, MI USA; 3 Department of Neurosurgery, University of Michigan, Ann Arbor, MI USA; 4 Department of Psychiatry, University of Michigan, Ann Arbor, MI USA; 5 Department of Biomedical Engineering, University of Michigan, Ann Arbor, MI USA

**Keywords:** Phonemic fluency, Semantic fluency, Subthalamic nucleus, Deep brain stimulation, Parkinson disease, Volume of tissue activation

## Abstract

Deep brain stimulation (DBS) of the subthalamic nucleus (STN) effectively treats Parkinson disease (PD) motor symptoms. However, STN DBS commonly leads to declines in verbal fluency (VF). This study aimed to identify STN stimulation regions that optimize motor benefits while minimizing negative impacts on phonemic (PVF) and semantic verbal fluency (SVF). 40 PD patients who underwent bilateral STN DBS (72 implants) were retrospectively analyzed. Individualized volume of tissue activation (VTA) models were utilized to comprehensively assess stimulation spread and STN activation. Regional VTA-STN overlap and external activation, as well as active contact position, were correlated with PVF and SVF outcomes using regression and Wilcoxon tests to determine the effect of stimulation location on VF. Motor speech outcome was also evaluated. Patients significantly declined in both PVF (*p* = 0.0001) and SVF (*p* = 0.008) post-DBS. PVF decline was significantly associated with more activation in the dorsal region of the left STN (*p* = 0.031). SVF decline correlated with significantly less activation in the anterior region outside the right STN (*p* = 0.036) and more posterior active contacts relative to the right STN centroid (*p* = 0.042). No significant impact of DBS on motor speech outcome or its relationship to stimulation location was observed. Utilizing individualized VTA models provided insights into specific stimulation regions potentially affecting VF. These findings underscore the importance of precise STN DBS targeting in surgical planning and clinical programming to balance motor benefits with the preservation of cognitive speech functions in PD patients.

## Introduction

Parkinson disease (PD) is a neurological movement disorder that affects both motor and non-motor functions. Common motor symptoms of PD include: muscular rigidity, slowness of movement, and involuntary tremors, while common non-motor symptoms include: cognitive impairment, pain, depression, and sleep disturbances (Yang et al. 2016; Hariz and Blomstedt 2022). Deep brain stimulation (DBS) is primarily used to treat PD motor symptoms. The treatment involves surgically implanted electrodes that stimulate specific structures deep in the brain with pulses of electricity controlled by a neuropacemaker (Hariz and Blomstedt 2022). Although stimulation of the subthalamic nucleus (STN), a standard target for PD, relieves motor symptoms, it can also cause cognitive side effects (Smeding et al. 2006; Floden et al. 2018).

After undergoing STN DBS, PD patients sometimes experience speech- and language-related complications, including declines in verbal fluency (VF) (Smeding et al. 2006; Le Goff et al. 2015; Wyman-Chick 2016; Floden et al. 2018; Greif et al. 2021; Askari et al. 2022). The MDS-Unified Parkinson’s Disease Rating Scale (MDS-UPDRS) is a clinical scale to evaluate the severity of PD symptoms, including speech (item 3.1), which refers to the motor ability to speak clearly or without stammering (Goetz et al. 2008). However, the MDS-UPDRS does not assess cognitive aspects of speech like VF. The Controlled Oral Word Association Test (COWAT) and Category Fluency Test (CFT) do, assessing phonemic and semantic fluency, respectively (Greif et al. 2021). Semantic verbal fluency (SVF) is evaluated by allowing patients one minute to verbalize words that are representative of a specified category (e.g., animals) (Isaacs and Kennie 1973). Phonemic verbal fluency (PVF) is evaluated similarly, but for words that begin with a specific letter (e.g., C, F, and L) (Patterson 2011).

To identify an optimal stimulation location that alleviates motor symptoms without negatively impacting VF, previous studies have investigated the relationship between electrode position and VF outcome following STN DBS (York et al. 2009; Witt et al. 2013; John et al. 2021; Greif et al. 2021). Electrode positions associated with VF declines vary across such studies, which may be explained by the metric used to evaluate stimulation location. The position of the electrode does not account for current spreading through the tissue from the active contact. This limitation can be addressed by computing the volume of tissue activation (VTA) around the electrode (Åström et al. 2010; Mikos et al. 2011; Malaga et al. 2021). Modeling the VTA can offer insights into specific regions within and around the STN that affect VF when activated, allowing for a comprehensive evaluation of stimulation location. VTA models can also be tailored to individual patients by incorporating their clinical neuroimaging and stimulation parameters, potentially providing more accurate and relevant results (Malaga et al. 2021). Therefore, the main objective of this study was to identify regions of activation associated with minimal changes in VF outcomes, specifically PVF and SVF, utilizing individualized VTA models built from PD patients who previously received STN DBS.

## Methods

### Patients and clinical data

In this retrospective study, 40 idiopathic PD patients (sex: 28 male, 12 female; handedness: 36 right, 4 left) who had undergone bilateral STN DBS at the University of Michigan were included from a prospectively maintained clinical database (Table 1). Details regarding the surgical procedure and inclusion criteria have been previously reported (Patil et al. 2012; Malaga et al. 2021). All patients were implanted with Medtronic systems, specifically 37 Activa PC and three Kinetra implanted pulse generator (IPG) models. One quadripolar lead (Medtronic Model 3389 **RRID: SCR_003988**) was implanted in each STN. However, eight implants were excluded from subsequent analysis due to non-monopolar stimulation programming post-surgery, resulting in 72 implants and 72 STNs (hemisphere: 37 left, 35 right) across the 40 patients. Informed consent was obtained from all patients and Institutional Review Board approval was obtained from Bucknell University and the University of Michigan to use the clinical data for the purposes of this study.

VF outcomes were evaluated using the COWAT and CFT for PVF and SVF, respectively (Greif et al. 2021). Pre- and post-operative raw VF scores were acquired in the on medication state for 30 of the 40 patients. VF change was defined as the difference between the post- and pre-operative scores divided by the pre-operative score, such that a positive value indicated improvement: VF change = (VF score_post−DBS_ − VF score_pre−DBS_)/VF score_pre−DBS_ × 100.

Motor speech outcome was evaluated using item 3.1 of the MDS-UPDRS (Chou et al. 2013). Post-operative speech scores were acquired off and on stimulation in the off medication state for all patients. Speech change was defined similarly to VF change, except not as a percentage: speech change = speech score_off DBS_ − speech score_on DBS_.

The clinical outcome scores were recorded as axial metrics. Consequently, left and right implants were assigned the same scores for each patient (if applicable) (Zak et al. 2025).

### Individualized VTA modeling

The STN and VTA data were acquired previously in a study that described the N-of-1 tissue activation modeling approach (Malaga et al. 2021). Briefly, anatomical models of the STN were derived from pre-operative magnetic resonance imaging (MRI). The STN borders were manually traced on coronal MRI slices using Analyze (Analyze Software System Version 12.0 **RRID: SCR_005988**). VTA models incorporated lead and electrode location and anisotropic tissue conductivity, derived from post-operative computed tomography (CT) and pre-operative diffusion tensor imaging (DTI), respectively, as well as therapeutic voltage amplitude. The electrode coordinates were measured on CT slices in all views using Analyze and the conductivity tensors were estimated from DTI eigenvalue and eigenvector maps (Tuch et al. 2001) using Analyze and MATLAB (MATLAB Version R2021a **RRID: SCR_001622**). The electric fields induced by DBS were computed using finite element analysis after integrating the imaging-based model components in COMSOL (COMSOL Multiphysics Version 5.2 **RRID: SCR_014767**). The VTAs were defined by thresholding the electric field norm at stimulation-specific activation levels (Åström et al. 2015). For each patient, associated with each STN-implant pair was an individualized VTA model. Given that the majority of patients had the same IPG type, the stimulation system heterogeneity was considered minimal and, therefore, unlikely to have a systematic impact on the derived tissue activation volumes. To measure stimulation location relative to the DBS target, the STNs and VTAs were converted to point cloud data.

### Stimulation location analysis

Stimulation location was characterized based on the position of the VTA and the active contact relative to the STN (Zak et al. 2025). In the VTA-based approach, the overlap between the VTA and the STN was measured by calculating a boundary around the VTA point cloud, counting the number of voxels comprising the STN point cloud inside the VTA boundary, and dividing that number by the total number of STN voxels. STN activation was further characterized by labeling the STN voxels according to their position relative to the centroid of the STN in the lateral-medial (X), anterior-posterior (Y), and dorsal-ventral (Z) directions (e.g., voxel above centroid = *dorsal*, voxel below centroid = *ventral*). This allowed regional STN activation to be measured as well (e.g., dorsal STN activation).

External activation, defined as the part of the VTA that did not overlap with the STN, was measured in a similar manner, after mapping the VTA boundary to a grid of voxels that matched the structure of the STN point cloud (0.5 × 0.5 × 0.5-mm^3^ voxels). Here, a boundary around the STN point cloud was calculated, the number of voxels comprising the restructured VTA point cloud outside the STN boundary was counted, and that number was divided by the total number of VTA voxels. Regional external activation was also measured similarly.

In the electrode-based approach, the position of the active contact relative to the STN centroid was measured in the X, Y, and Z directions. To account for anatomical differences in STN size across patients and hemispheres, the electrode coordinates were inversely scaled by the appropriate dimension of the STN, defined as the absolute difference between the maximum and the minimum STN coordinate in a given direction (e.g., height = |Z coordinate_max_ − Z coordinate_min_|). All of the stimulation location analysis was done using MATLAB.

### Statistical analysis

The effect of STN DBS on VF (30 patients) and on motor speech (40 patients) was evaluated using the paired, two-sided Wilcoxon signed rank test.

To investigate the influence of lateralization, the implants were separated by hemisphere (Greif et al. 2021). The implants were then grouped based on VF and motor speech outcomes. Because VF decline was considered a side effect, an implant was put into the *Zero/Positive* group if its associated VF change was greater than or equal to zero; else it was put into the *Negative* group. For speech, a motor symptom, an implant was put into the *Positive* group if its associated speech change was greater than zero; else it was put into the *Zero/Negative* group. After grouping the implants, the VTA- and electrode-based stimulation location metrics associated with them were compared between the two groups using the two-sided Wilcoxon rank sum test. This was done separately for both sides.

The general relationship between clinical outcomes and stimulation location was evaluated using stepwise regression and Pearson’s correlation, with the stimulation location metrics as the predictors (15 metrics) and the VF and motor speech changes as the response variables (three outcomes, one model per outcome).

All of the statistical analysis was done using MATLAB. Significance was defined at a p-value less than 0.05. Values separated by “±” indicate the mean and standard deviation (SD), respectively.

## Results

### Patient demographics and clinical stimulation settings

The demographics of the PD patients included in this study, along with their therapeutic stimulation settings from the last follow-up appointment (6 − 24 months), are provided in Table 1.

**Table 1:** Patient demographics^a^ and clinical stimulation settings^b^

	Mean ± SD	Min − Max
Age, baseline (yr)	63.1 ± 6.7	52.7−74.7
Age, diagnosis (yr)	52.8 ± 8.4	33.0−68.0
Disease duration (yr)	10.3 ± 5.2	2.0−22.8
Voltage, left contact (V)	2.7 ± 0.6	1.5−4.2
Frequency, left contact (Hz)	143.1 ± 24.1	125−185
Pulse width, left contact (µs)	60.8 ± 4.9	60−90
Voltage, right contact (V)	2.6 ± 0.6	1.5−3.9
Frequency, right contact (Hz)	139.3 ± 21.1	125−185
Pulse width, right contact (µs)	60.0 ± 0.0	60−60

^a^n_patient_ = 40

^b^Measured at active contact; n_contact, left_ = 37, n_contact, right_ = 35

### VF outcomes and their relationship with stimulation location

**Fig. 1:**
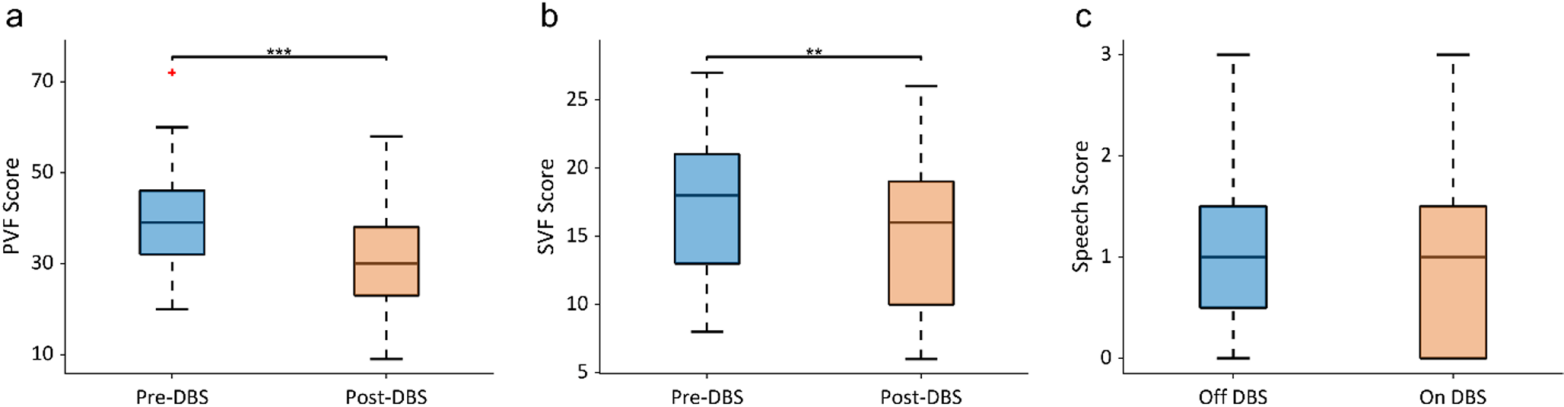
**a** PVF and **b** SVF scores from the COWAT and CFT, respectively, before and after DBS surgery (*n* = 30). ** *p* < 0.01 and *** *p* < 0.001. **c** Motor speech scores from the MDS-UPDRS (item 3.1) with DBS off and on after surgery (*n* = 40)

The PVF and SVF scores for all patients who had them recorded (30/40 patients) are summarized in Fig. 1a, b. PVF score decreased significantly from 40.0 ± 12.0 before DBS to 31.7 ± 12.2 after DBS (*p* = 0.0001), corresponding to a −20.0 ± 21.9% change. SVF score also decreased significantly, albeit to a lesser degree, from 17.6 ± 5.2 before DBS to 15.0 ± 4.8 after DBS (*p* = 0.008), corresponding to a −11.2 ± 30.1% change.

**Fig. 2:**
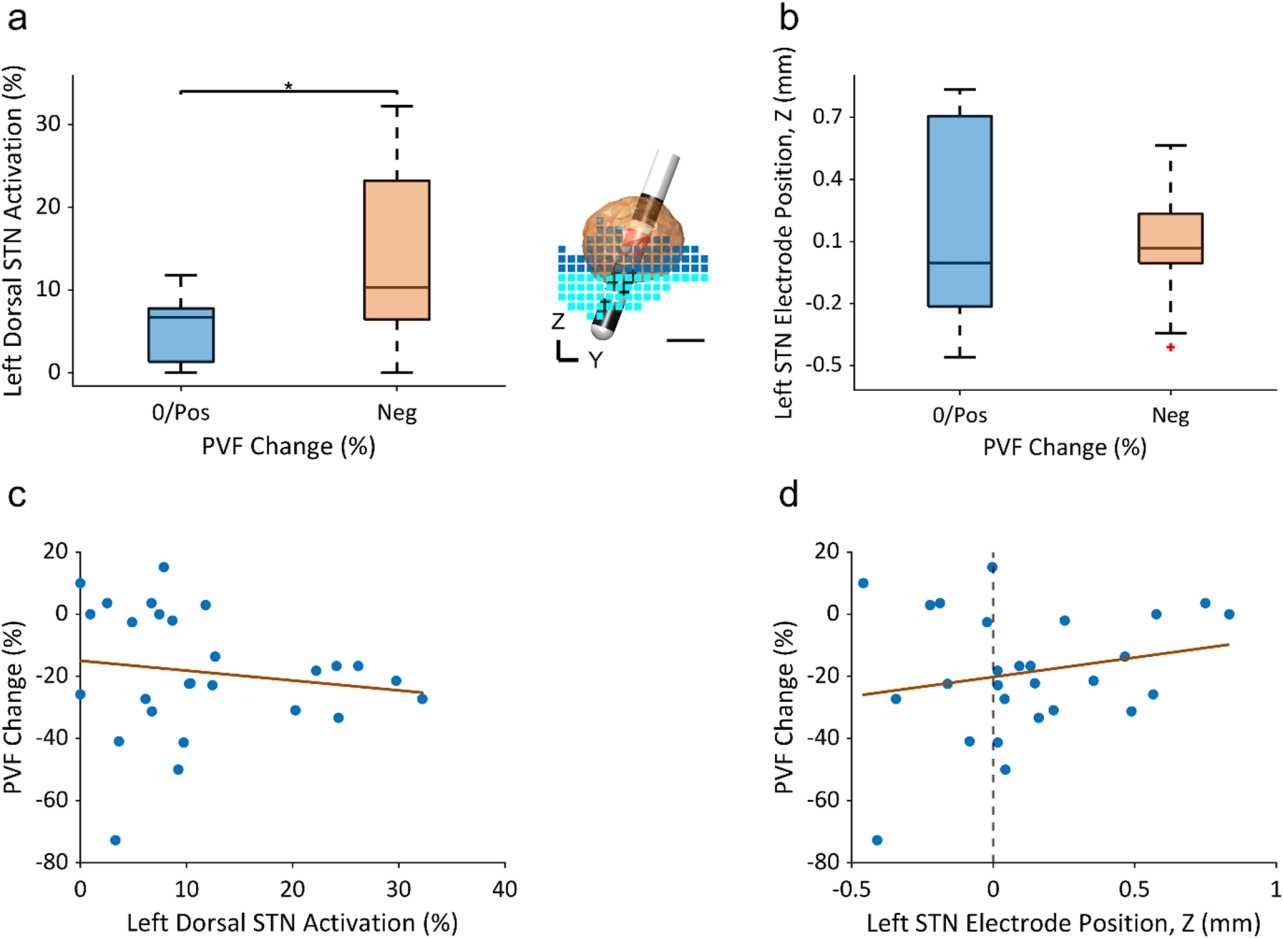
**a** Comparison of dorsal STN activation in the left hemisphere based on PVF change. The Zero/Positive and Negative groups contain 7 and 20 implants, respectively. * *p* < 0.05. Inset shows the left STN (dorsal region = blue, ventral region = cyan) and majority dorsal VTA (orange) of an exemplar patient. Scale bar is 2 mm, sagittal view. **b** Electrode Z position in the left hemisphere comparison based on PVF change. Same grouping as in panel a. **c** Linear relationship between dorsal STN activation in the left hemisphere and PVF change for 27 implants. **d** Electrode Z position in the left hemisphere-PVF change relationship for the same implants as in panel c. Dashed line indicates the Z position of the STN centroid

Regarding stimulation location, patients with a negative PVF change had significantly more dorsal STN activation in the left hemisphere than those with a zero or positive change (*p* = 0.031) (Fig. 2a). However, the two groups had no significant difference in electrode position in the Z direction (Fig. 2b). The general trend between dorsal STN activation in the left hemisphere and PVF change was negative (Fig. 2c), while electrode position in the Z direction trended positively with PVF change (Fig. 2d). Neither relationship was significant though. No significant findings were observed in the right hemisphere.

**Fig. 3:**
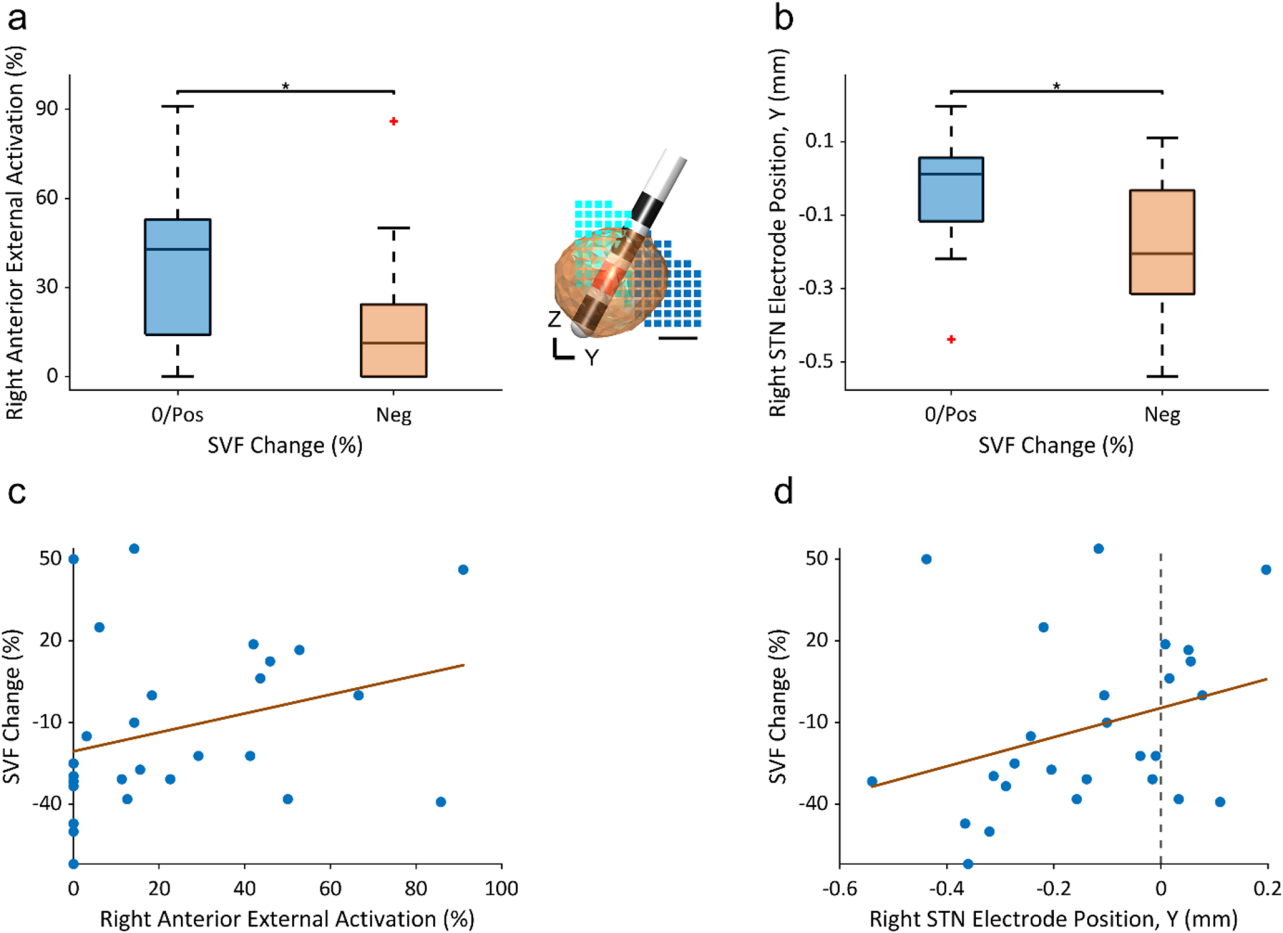
**a** Comparison of anterior external activation in the right hemisphere based on SVF change. The Zero/Positive and Negative groups contain 10 and 17 implants, respectively. * *p* < 0.05. Inset shows the right STN (anterior region = blue, posterior region = cyan) and majority posterior VTA (orange) of an exemplar patient. Scale bar is 2 mm, sagittal view. **b** Electrode Y position in the right hemisphere comparison based on SVF change. Same grouping as in panel a. **c** Linear relationship between anterior external activation in the right hemisphere and SVF change for 27 implants. **d** Electrode Y position in the right hemisphere-SVF change relationship for the same implants as in panel c. Dashed line indicates the Y position of the STN centroid

In the right hemisphere, patients with a negative SVF change had significantly less anterior external activation than those with a zero or positive change (*p* = 0.036) (Fig. 3a). Furthermore, the two groups had a significant difference in electrode position in the Y direction, where SVF decline was associated with more posterior active contacts (*p* = 0.042) (Fig. 3b). The same relationships were observed in the left hemisphere, but they were not significant. The general trend between anterior external activation in the right hemisphere and SVF change was positive (Fig. 3c). Electrode position in the Y direction also trended positively with SVF change (Fig. 3d). However, neither relationship was significant. This was the case in the left hemisphere as well.

### Motor speech outcome and its relationship with stimulation location

The motor speech scores for all patients are summarized in Fig. 1c. Unlike the VF outcomes, speech score did not differ significantly after surgery when DBS was off and on (*p* = 0.183). Speech score was 1.1 ± 0.9 off DBS and 0.9 ± 0.9 on DBS, corresponding to a 0.2 ± 0.6 change. Furthermore, no significant relationship between stimulation location (VTA- and electrode-based) and speech outcome was observed in either hemisphere.

The general trend between speech change and VF (PVF and SVF) change was negative, but not significant. To expand motor outcome beyond a single MDS-UPDRS item, speech change was substituted with the change in cardinal motor symptoms (rigidity, bradykinesia, and tremor) for the patients (Malaga et al. 2021). This revealed a significant relationship with SVF change in the left hemisphere (*r* = −0.52, *p* = 0.005, *n* = 27) that was consistent with the trend between speech and VF. Unlike speech, the motor symptom scores were lateralized. In the left hemisphere, motor symptom score was 14.1 ± 6.6 off DBS and 7.5 ± 4.9 on DBS, corresponding to a 41.9 ± 54.6% change (*n* = 37). Motor symptom score was 15.4 ± 6.6 off DBS and 9.1 ± 5.8 on DBS, corresponding to a 38.6 ± 30.7% change, in the right hemisphere (*n* = 35).

## Discussion

Consistent with previous studies (Smeding et al. 2006; Mikos et al. 2011; Mossner et al. 2020; John et al. 2021; Greif et al. 2021; Askari et al. 2022; Schoenwald et al. 2025), despite receiving motor symptom relief, patients as a cohort experienced declines in VF after undergoing STN DBS. This study sought to expand on current understanding of the relationship between stimulation location in the subthalamic region and VF, as well as motor speech, outcomes using an individualized VTA modeling approach (Malaga et al. 2021). The main results revealed that PVF decline was associated with more stimulation in the dorsal region of the left STN, while SVF decline was associated with less stimulation in the anterior region outside the right STN.

Findings in the literature are mixed regarding the relationship between stimulation site and VF outcomes. The association between PVF decline and dorsal STN activation in the left hemisphere aligns with John et al., who found that VF decline was greatest when active contacts were located more dorsally in the left STN region (John et al. 2021). However, differences in their methods include using electrode position as a stimulation location metric and overall fluency (average of semantic, phonemic, and action fluency) change as a VF outcome metric. This result is also consistent with Mossner et al., who identified the site of maximal PVF worsening to be dorsal, medial, and posterior to the STN midpoint (Mossner et al. 2020). Instead of electrode position, they used a computational electrical field model to determine the stimulation site. Although this approach is conceptually similar to VTA modeling, it is primarily based on an activation function representing the probability of neuronal activation. Furthermore, they did not differentiate by hemisphere. Mikos et al. reported seemingly contradictory results, finding that decreased PVF was associated with more STN activation at ventral contacts and, conversely, that improved PVF was associated with more STN activation at optimal contacts (generally dorsolateral, where motor benefits are maximized) (Mikos et al. 2011). Despite using VTA modeling to characterize stimulation location, methodological differences, such as defining ventral contacts relative to optimal contacts, grouping left and right hemispheres together, and incorporating electrical tissue properties from a DTI brain atlas (Liu et al. 2024), may explain the discrepancy in these results. Notably, their patients underwent unilateral STN DBS. Greif et al. found that more anterior contacts relative to the left STN midpoint predicted greater PVF decline (Greif et al. 2021), while Floden et al. reported that PVF worsened with more posterior contacts in the left STN relative to the midcommissural point (MCP) (Floden et al. 2018). Both of their results identified different (and opposite) associated locations, highlighting an anterior-posterior impact rather than a dorsal-ventral impact. This discrepancy could arise from differences in how stimulation location was measured (electrode position versus VTA location and STN midpoint versus MCP). A potential reconciliation between these results is that current from an anterior or posterior contact can spread to the dorsal STN region. This is precisely the advantage that VTA modeling provides: it allows the spread of stimulation from the active contact in all directions to be evaluated. While this study focused on stimulation location, other research has highlighted the importance of the surgical trajectory (Zahodne et al. 2009; York et al. 2009; Witt et al. 2013; Le Goff et al. 2015; Askari et al. 2022). Askari et al. found that lateral penetration of the superior frontal gyrus in the left hemisphere predicted greater PVF decline, suggesting microlesioning effects on language pathways may be a stronger predictor than electrode position (Askari et al. 2022) and perhaps VTA location.

In the right hemisphere, the association between SVF decline and anterior external activation, as well as posteriorly located electrodes, was unexpected because previous studies have reported SVF decline to be lateralized to the left hemisphere like PVF (Zahodne et al. 2009; Witt et al. 2013; Le Goff et al. 2015; Floden et al. 2018; Zhao et al. 2025). Although this relationship was the same in the left hemisphere, it was not significant. Regardless, this result partially aligns with York et al., who found that SVF decline was only related to contacts located more dorsally in the right STN region (York et al. 2009). They used an electrode position-based approach, so stimulation spread from the dorsal contacts in the anterior-posterior direction cannot be readily compared. Furthermore, their anatomical reference point was the MCP. Despite reporting that unilateral left-sided STN DBS surgery was associated with SVF decline, Zahodne et al. also found that right-sided surgery still had a risk of decline, albeit lower (Zahodne et al. 2009). Tröster et al. investigated VF outcomes after unilateral pallidal surgery (globus pallidus internus DBS or pallidotomy), finding no laterality effect for SVF decline as it was observed on both sides and stating that SVF performance is not consistently lateralized to the left hemisphere (Tröster et al. 2002). Although not a DBS outcome study, Khateb et al. stated that the right hemisphere also has a role in processing semantic information (Khateb et al. 2001), providing conceptual support to the laterality of this result. Greif et al. reported that electrode position did not predict SVF decline in either hemisphere (Greif et al. 2021). As discussed above, this discrepancy may be explained by differences in methods of measuring stimulation location. The association between SVF decline and right hemisphere stimulation locations supports the view that SVF is a distributed, network-level function and is not consistently lateralized to the left hemisphere (Tröster et al. 2002). While the assumption of a primarily left-dominant language system is common (Lin et al. 2021), the right hemisphere plays a role in processing semantic information (Khateb et al. 2001). The distinct lateralized effects for PVF and SVF found in this study provide a more nuanced interpretation of right-left differences. Further research into right hemisphere involvement in SVF decline after STN DBS is warranted.

Motor speech change, in contrast to the declines in cognitive speech-related outcomes, was not associated with stimulation location. Furthermore, DBS had no significant impact on speech. These results align with Kim et al., who generally found no-to-minimal speech change between DBS on and DBS off based on perceptual ratings (Kim et al. 2024). Although some acoustic measures showed differences, they acknowledged that significance was driven by a few participants as most did not exhibit consistent trends. The lack of a DBS effect on speech is also consistent with Gessani et al., who found that speech intelligibility was maintained five years after surgery (Gessani et al. 2023). Regardless, speech decline after STN DBS is commonly reported despite outcomes often being heterogeneous and variable (Dromey and Bjarnason 2011; Fabbri et al. 2021; Tabari et al. 2024). Kluin et al. found that dysarthria rating scores worsened significantly from mild to mild-moderate impairment (Kluin et al. 2022). Using the same computational electrical field approach as Mossner et al., they identified the site of dysarthria worsening to be ventral and anterior to the STN midpoint, distinct from the (dorsal and posterior) maximal motor improvement site. Åström et al. also observed impaired speech intelligibility (Åström et al. 2010). However, their VTA analysis linked this decline to stimulation spread from electrodes positioned medial and/or posterior to the STN centroid. Methodological differences in speech assessment, as well as stimulation location measurement differences, likely contributed to the discrepancy in these results. More sensitive and comprehensive speech assessment tools can capture the nuanced changes that item 3.1 of the MDS-UPDRS cannot.

From a clinical perspective, the dorsal STN, which is an optimal stimulation region for motor symptom improvement (Malaga et al. 2021), is also the region most associated with PVF decline (Mossner et al. 2020). Because these stimulation regions are in close proximity, identifying a “sweet spot” that provides robust motor benefit without any negative impact on VF is challenging. This finding is not intended to influence surgical targeting, but instead can be used to inform the personalization of postoperative programming and manage patient expectations. Furthermore, it remains difficult to predict with certainty which region(s) will receive the most stimulation in individual patients due to the complex interplay of factors affecting the spatial extent of activation, including stimulation parameters and tissue impedance (Malaga et al. 2021; Kumar et al. 2024).

Several limitations should be considered when interpreting the results of this study. First, the speech item on the UPDRS (and implicitly the MDS-UPDRS), which rates overall speech function on a 0 − 4 scale, has shown poor interrater reliability compared to the other motor items (Richards et al. 1994). Due to its low resolution and high subjectivity, this assessment may be insufficient for capturing subtle speech changes (Kim et al. 2024). Additional, more objective speech measures, such as acoustic features (Dromey and Bjarnason 2011; Kim et al. 2024), could be used in complementary analyses to investigate stimulation location-speech outcome relationships. Second, the STN was the only brain structure segmented. This limited the external activation metric to regional characterizations of non-STN stimulation location. Because stimulation usually spreads beyond the STN (Malaga et al. 2021), the activation of nearby structures, such as the internal capsule (Kluin et al. 2022; Liu et al. 2024) and the fasciculus cerebellothalamicus (Åström et al. 2010), could be computed instead of a generic activation metric to perform a more detailed VTA analysis. Third, the sample size was relatively small, potentially limiting the ability to detect more nuanced effects. Although dividing the data by hemisphere was deemed necessary due to the reported lateralization of speech-related outcomes (Lin et al. 2021), doing so reduced the sample size and statistical power. This is a common challenge in studies comparing lateralized DBS effects (Woods et al. 2006; Lin et al. 2021), particularly in retrospective studies (such as this) as they lack full experimental control. Prospective studies with larger cohorts of patients, as well as measurement of clinically relevant outcomes, are needed to determine if these results are generalizable such that they can inform decision-making when optimizing STN DBS parameters. Last, additional demographic information was not available, such as education and language dominance, which could affect VF outcomes and lateralization and should be assessed in future studies.

## Conclusion

This study examined how stimulation location within and around the STN affects VF outcomes in 40 PD patients who underwent bilateral STN DBS. Using individualized VTA models, stimulation spread from therapeutic contacts was assessed. Patients showed significant declines in both PVF and SVF after DBS. PVF decline was significantly associated with more stimulation in the dorsal left STN, while SVF decline was linked to less stimulation in the anterior region outside the right STN and more posterior contact locations relative to the right STN centroid. In contrast, no significant effects were observed for motor speech or its relationship to stimulation location. These findings underscore the importance of precise targeting in STN DBS to balance motor improvement with preservation of verbal fluency.

## Data Availability

The data that support the findings of this study are available from the corresponding author upon reasonable request. Due to the clinical nature of the data and privacy considerations, the dataset cannot be shared openly.
